# Non-islet Cell Tumor-induced Hypoinsulinemic Hypoglycemia in the Setting of Metastatic Desmoplastic Round Cell Tumor

**DOI:** 10.7759/cureus.4669

**Published:** 2019-05-15

**Authors:** Nadir Bhuiyan, Purva Sharma

**Affiliations:** 1 Internal Medicine, University of Miami, Miller School of Medicine, Atlantis, USA

**Keywords:** paraneoplastic syndrome, tumor induced hypoglycemia, desmoplastic round cell tumor, non-islet cell tumor induced hypoglycemia

## Abstract

A 24-year-old male with metastatic desmoplastic round cell tumor was admitted for fatigue and weakness after chemotherapy. The patient was found to be hypotensive, pancytopenic, and bacteremic. Early treatment with intravenous antibiotics and fluids was efficacious. The hospital course was complicated by recurrent hypoglycemia that was refractory to the standard hypoglycemia protocol. Initial workup revealed low serum insulin and normal C-peptide. Further evaluation revealed elevated IGF-II levels consistent with non-islet cell tumor-induced hypoglycemia. Euglycemia was subsequently achieved with aggressive and continuous infusion of intravenous 10% dextrose.

## Introduction

Non-islet cell tumor hypoglycemia (NICTH) is a rare life-threatening paraneoplastic syndrome that can be precipitated by a wide variety of malignancies. In this condition, tumors characteristically secrete detectable hormones, or hormone-like substances that facilitate persistent, rapid declines in blood glucose levels. While there are myriad physiologic pathways that result in tumors causing hypoglycemia, one of the better understood mechanisms is through secretion of insulin-like growth factor II (IGF-II) and its precursor protein. We describe the diagnosis and management of IGF-II mediated of hypoinsulinemic refractory hypoglycemia in the setting of diffuse, metastatic desmoplastic round cell tumor.

## Case presentation

**Figure 1:**
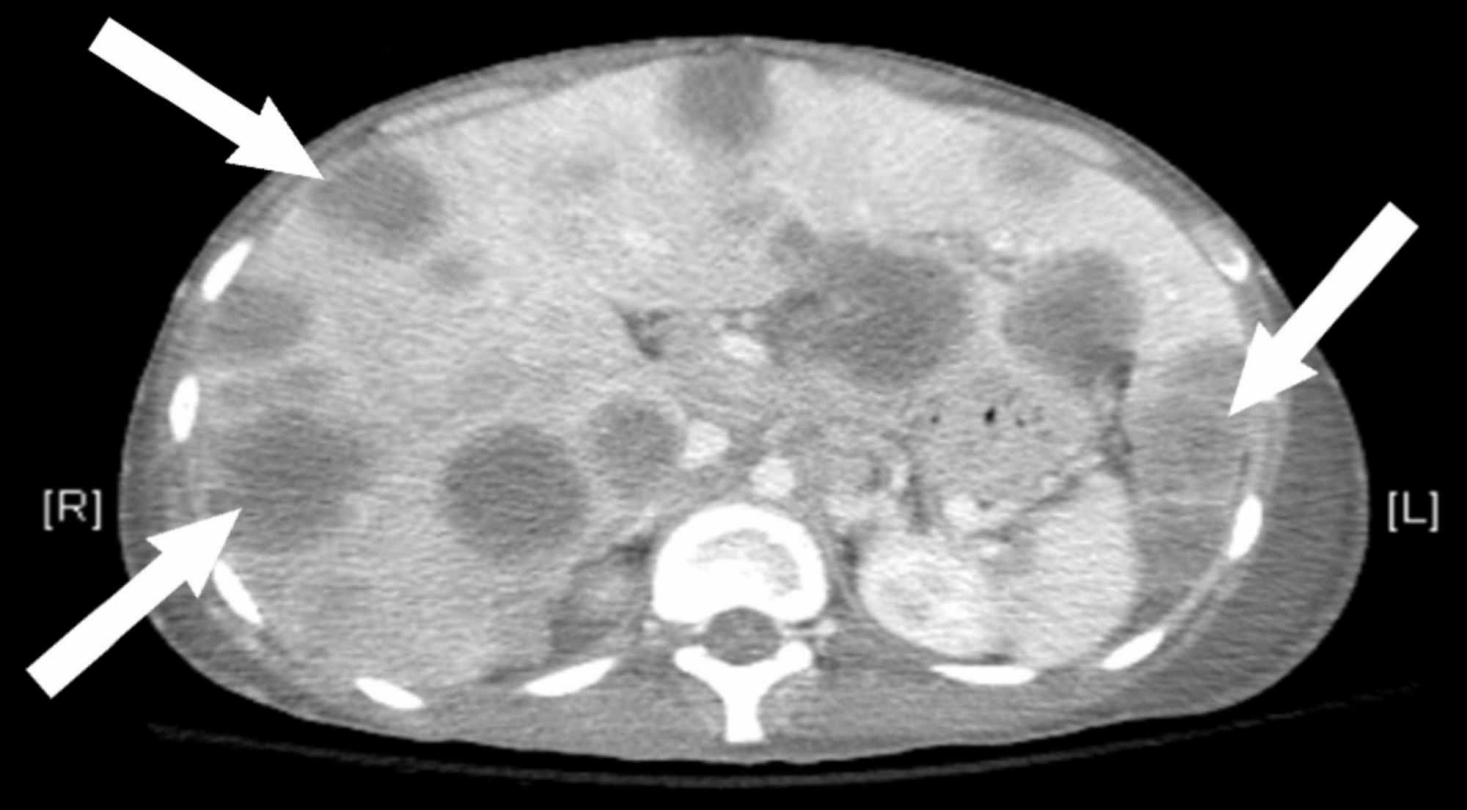
Axial CT scan of the abdomen with intravenous contrast demonstrating innumerable liver metastases. *Arrows demonstrating significant metastatic tumor burden in the liver with massive hepatomegaly.

A 24-year-old Hispanic male with recently diagnosed desmoplastic round cell tumor with innumerable metastases, presented with complaints of significant weakness and fatigue after receiving one cycle of vincristine, adriamycin, and ifosfamide (VAI). Eleven days after his first cycle of chemotherapy, he presented to the emergency department with complaints of subjective fever, weakness, and worsening fatigue. He was found to be hypotensive with pancytopenia and was subsequently admitted to the intensive care unit (ICU) for further management. Physical examination was notable for cachexia and rigid hepatomegaly. Both blood cultures were positive for *Streptococcus dysgalactiae*. CT scan of the abdomen and pelvis with IV contrast revealed impressive metastatic infiltration into the liver (Figure 1). The patient's condition rapidly improved with the administration of intravenous fluids and vancomycin. Filgrastim was added to address the neutropenia. On day two of his hospital course, he developed severe hypoglycemia. Initial treatment included boluses of 50% dextrose solution; after two administrations the patient remained persistently hypoglycemic. Frequent small meals with high sugar content provided negligible benefit. Management was escalated to a continuous infusion of 5% dextrose solution and ultimately to 10% dextrose before adequate control of blood glucose was achieved. With no clear cause of hypoglycemia, the tumor was suspected as the etiology. Within 48 hours of intensive correction and monitoring, his blood glucose was stabilized. To differentiate between islet cell tumor-induced hypoglycemia and non-islet cell tumor-induced hypoglycemia (NICTH), the preliminary tests ordered were insulin and C-peptide levels. With a normal C-peptide level and low insulin level, further hormonal causes were explored. Cortisol levels were elevated, likely due to systemic stress response to prior infection and hypoglycemia. Thyroid stimulating hormone (TSH) and thyroxine (T4), were within their normal reference ranges. Growth hormone was elevated (Table 1). The possibility of an auto-immune paraneoplastic process was investigated by searching for insulin antibodies; the results were negative. Insulin-like growth factors I and II (IGF-I, IGF-II) are recognized mediators of NICTH, specifically IGF-II [1]. These polypeptides share structural similarity to pro-insulin revealing, at least in part, the physiologic basis of their effect on serum blood glucose. The ratio of IGF-II to IGF I is used as a diagnostic value in NICTH. A ratio of 10 or greater is the conventional pathognomonic value, 3 is considered the standard baseline [1-4]. IGF-II levels were pointedly elevated in comparison to IGF-I levels, making the ratio indicative of NICTH (Table 1). The patient's blood glucose level was maintained for two weeks but he was not able to tolerate any further chemotherapy. A recurrence of septic shock leads to the development of multi-organ failure. Unfortunately, the patient expired 15 days after admission to the ICU.

**Table 1:** Serum laboratory values. GH: Growth hormone; IGF: Insulin-like growth factor.

Serum Test	Laboratory Value	Reference Ranges
Insulin	1.2 mU/L	3-25 mU/L
C-Peptide	0.61 ng/mL	0.48-5.05 ng/mL
Insulin Antibodies	<5	> Or = 5 is positive
Cortisol	56.63 mcg/dL	3.09-16.66 mcg/dL P.M.
GH	16.7 ng/mL	0-3 ng/mL
IGF-I	38.8 mg/mL	116-358 ng/mL
IGF-II	4600 ng/nL	84-580 ng/mL
IGF-II/I Ratio	118.6	Diagnostic at minimal 2/1 ratio

## Discussion

Tumor-induced hypoglycemia is a serious paraneoplastic syndrome which can be divided into islet cell and non-islet cell sub-types. In regards to the beta (β) islet cell sub-type, insulinoma is the classic tumor of which 5-10% are malignant. The non-islet cell sub-type can be further divided into ectopic insulin producing tumors. These include endobronchial carcinoid, cervical squamous-cell carcinoma, neurofibrosarcoma, gastrointestinal stromal tumor (GIST), schwannoma, and paraganglioma [1]. The other division should include a category of insulin-independent hypoglycemia. The vast majority of these tumors secrete circulating factors that have homology with insulin or are insulin secretagogues.

The most frequently reported form of this is IGF-II mediated tumor-associated hypoglycemia. IGF-I and II share protein homology with insulin, capable of activating the receptor and mimicking insulin activity. As one would expect, hypoglycemia is a common consequence of this. Due to over-expression of the IGF-II gene in certain tumors, large quantities of IGF-II and Pro-IGF-II are secreted. Pro-IGF-II is the precursor protein produced by the gene, prior to enzymatic cleavage [4]. In the literature it is often referred to as 'Big IGF-II'. Of note, big IGF-II has more affinity to the insulin receptor, increasing the likelihood that hypoglycemia will manifest. The ratio of IGF-II to IGF-I is used as surrogate measurement for big IGF-II. The physiologic explanation of this is that GH and thus IGF-I are decreased due to negative feedback from high levels of IGF-II. A ratio greater than 10 is considered to be pathognomonic of IGF-II mediated NICTH. The implicated tumors include hepatocellular carcinoma, gastric carcinoma, solitary fibrous pleural tumor of the lung, desmoplastic small round cell tumor, mesothelioma and leiomyosarcoma, among a few others [2-3,5].

Somatostatinomas, typically found in close proximity with the duodenum and within the pancreas, have been implicated as well. These rare neuroendocrine tumors (NETs) excessively secrete somatostatin. The net effect of which impairs gastrointestinal fluid secretion and motility. Thus, a patient's presentation may include abdominal pain, steatorrhea, cholelithiasis, and diabetes mellitus. Glucagon and insulin, among other hormones, can be found at elevated levels due to the effects of overabundant somatostatin. Therefore, hypoglycemia in these tumors is not common but may involve suppression of both growth hormone and glucagon. Some NETs may produce glucagon-like peptide 1 (GLP) which results in secretion of insulin from pancreatic B-cells. Other mechanisms of note include antibodies that activate the insulin receptor, increased metabolic demand due to excessive tumor burden, extensive liver infiltration, and metastatic pituitary or adrenal gland invasion resulting in adrenal insufficiency [1].

Management of NICTH involves several methods of glucose correction. After rapid correction of hypoglycemia is achieved with IV dextrose, small and frequent meals should be administered. Ultimately, treatment of the tumor, whether by surgical resection or chemotherapy, should resolve the hypoglycemia. If this is not possible, then continuous infusions of intravenous dextrose may be used. For long-term management using corticosteroids, growth hormone, and glucagon, either individually or in various combinations, is recommended for maintenance of euglycemia [1-3, 5].

Desmoplastic small round cell tumor (DSRCT) is soft tissue sarcoma of mesenchymal origin. It presents as a rare aggressive type of cancer, usually affecting young males and located in the abdomen. It presents most often as a multifocal peritoneal malignancy with disseminated abdominal disease. It most commonly involves the omentum and peritoneum followed by the retroperitoneum. It may sometimes involve the kidney as well [6]. Patients may present with abdominal pain, abdominal mass, ascites, or signs of intestinal obstruction. The liver is the most common solid visceral metastatic site. It is also known to have involvement of the thorax including the pleura and lymph nodes. DSRCTs are characterized by chromosomal abnormalities. They present a reciprocal chromosomal translocation, t(11;22)(p13;q12), that results in fusion of Ewing's sarcoma (EWSR1) and Wilms' tumor (WT1) genes [7]. The fusion protein as a result of this translocation is pathognomonic for DSRCT. Treatment of DSRCT involves a multimodality approach involving chemotherapy, aggressive surgery, tumor debulking, and total abdominal radiation therapy. Overall, the patient response to these treatment modalities is poor and rarely achieves remission. There are only two cases reported in the literature of DSRCT directly manifesting hypoglycemia through big IGF-II [8-10], one in an adult and one in a pediatric patient.

We cannot exclude massive tumor burden or metastatic liver infiltration as a cause of hypoglycemia in our patient. However, we believe that these etiologies are less likely due to the refractory nature of the hypoglycemia in response to intravenous dextrose. This is more consistent with circulation factors that activate the insulin receptor, like big IGF-II.

## Conclusions

This case demonstrates a rare presentation of a paraneoplastic syndrome in a patient with a rare aggressive malignancy. NICTH is caused due to secretion of IGF-II or other related substances. Low levels of insulin or IGF-I in the setting of hypoglycemia should raise suspicion for NICTH. A focused workup including C-peptide levels and ruling out other endocrine causes of hypoglycemia is necessary. The diagnosis is confirmed with an IGF-II:IGF-I ratio greater than 10. Given the rarity of paraneoplastic syndromes there is a paucity of clinical trials to guide management. It is important to be aware of and recognize these syndromes as they can precede or occur at the time of tumor presentation or at a later stage. Continuous infusion of dextrose was successful in treating the hypoglycemia in our patient, however, newer treatment modalities such as anti-IGF-II therapies are being studied and could be an effective option in future.
